# Shape-Dependent Skin Penetration of Silver Nanoparticles: Does It Really Matter?

**DOI:** 10.1038/srep16908

**Published:** 2015-11-20

**Authors:** Yu Kyung Tak, Sukdeb Pal, Pravin K. Naoghare, Sabarinathan Rangasamy, Joon Myong Song

**Affiliations:** 1 College of Pharmacy, Seoul National University, Seoul 151-742, South Korea; 2 Council of Scientific and Industrial Research-National Environmental Engineering Research Institute (CSIR-NEERI), Nehru Marg, Nagpur 440020, India

## Abstract

Advancements in nano-structured materials have facilitated several applications of nanoparticles (NPs). Skin penetration of NPs is a crucial factor for designing suitable topical antibacterial agents with low systemic toxicity. Available reports focus on size-dependent skin penetration of NPs, mainly through follicular pathways. Herein, for the first time, we demonstrate a proof-of-concept study that entails variations in skin permeability and diffusion coefficients, penetration rates and depth-of-penetration of differently shaped silver NPs (AgNPs) via intercellular pathways using both *in vitro* and *in vivo* models. The antimicrobial activity of AgNPs is known. Different shapes of AgNPs may exhibit diverse antimicrobial activities and skin penetration capabilities depending upon their active metallic facets. Consideration of the shape dependency of AgNPs in antimicrobial formulations could help developing an ideal topical agent with the highest efficacy and low systemic toxicity.

Encouraging results have recently been reported regarding the bactericidal activity of silver nanoparticles (AgNPs) of either simple or composite nature^1^,^2^. Their application as topical antimicrobial agents to control colonization and proliferation of microbial pathogens including multidrug-resistant organisms is envisaged to revolutionize burn wound care therapy. AgNPs were shown to undergo size-dependent interactions with HIV-I and gram-negative bacteria^3^,^4^. In addition our group, for the first time, reported the comparative study on the bactericidal properties of silver nanoparticles of different shapes, and demonstrated that AgNPs also undergo shape-dependent interactions with bacteria^5^. Truncated triangular silver nanoplates with a {111} lattice plane as the basal plane displayed the strongest biocidal action, compared with spherical and rod-shaped nanoparticles and with Ag^+^. Surprisingly, though AgNPs were reported to penetrate through intact and damaged human skin, to the best of our knowledge, there is no report on shape- or size- dependent skin penetrations involving AgNPs. Skin permeation studies have revealed absorption of NPs through intercellular and follicular penetration pathways (Fig. 1A)^6^. In the intercellular pathway, solid NPs often prefer the tortuous route of penetration through corneocytes, arranged in the “brick and mortar” fashion. These arrangements hinder efficient transport of solid NPs through the layers of corneocytes (stratum corneum; 10–15 μm) embedded in the lipid matrix^7^,^8^. Intercellular heterogeneity, porosity of stratum corneum, dose/time dependency, and the lipid matrix also resist the intracellular penetration of NPs through stratum corneum^6^,^9^. This penetration pathway is not yet clearly understood. On the other hand, numerous reports agree that the follicular penetration pathway plays a central role in the penetration of NPs through non-damaged skin^6^,^10^,^11^,^12^. In order to comprehensively investigate these facts, spherical, rod-shaped and truncated triangular AgNPs were chemically synthesized, and their skin penetration capabilities were studied using *in vitro* and *in vivo* cell models. Herein, we report for the first time shape dependent skin penetration of AgNPs.

## Results

### Morphologies of AgNPs

**Figure 1:**
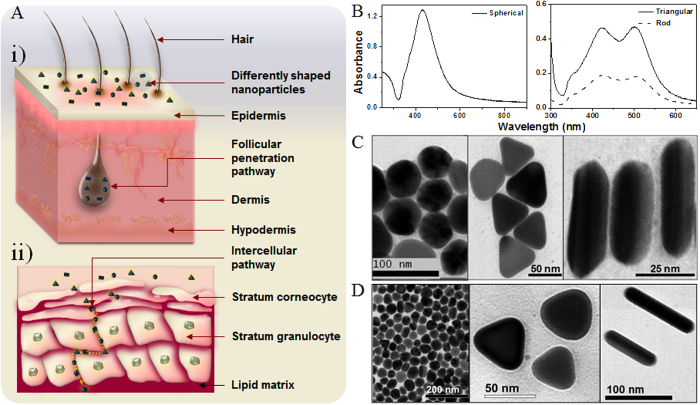
The schematic diagram of possible skin penetration pathways of three differently shaped AgNPs. (**A**) Two main possible skin penetration pathways are illustrated: (i) enters via hair follicles (the follicular penetration pathway); and (ii) diffuses through the gaps between corneocytes (the intercellular penetration pathway). (**B**) Absorption spectra of solutions containing chemically synthesized SNP, TNP, and RNP, showing different peaks of absorbance at certain wavelengths. (**C**) TEM images showing morphology of synthesized AgNPs. (**D**) TEM images clearly showing the discrete existence of differently shaped AgNPs after dispersing them in phosphate buffer (pH 7.0), the medium used in the donor compartment of the Franz cell. Dimensions of the AgNPs are almost similar to that of the synthesized ones.

Spectrophotometric analysis showed distinct absorbance spectra for differently shaped AgNPs (Fig. 1B)^5^. Average dimensions of the differently shaped AgNPs were determined from the transmission electron microscopic (TEM) image analysis (Fig. 1C). The average diameter of the spherical nanoparticles (SNPs) was 50 nm, whereas average length and diameter of rod-shaped nanoparticles (RNPs) were 50 and 20 nm, respectively. Triangular nanoparticles (TNPs) appeared as 2 nm thick equilateral triangular plates with average side length of 50 nm.

### Colloidal stability of AgNPs

Colloidal stability of the nanoparticles in the donor cell medium (phosphate buffer; pH 7.0) of the Franz cell system used for *in vitro* analysis was initially confirmed by measuring zeta potential of the AgNPs after dispersing them in phosphate buffer (pH 7.0). The measured zeta potentials of the AgNPs dispersed in distilled water as well as in phosphate buffer were all greater than 20 mV (Table 1) indicating that the NPs form stable colloids in phosphate buffer (pH 7.0) medium. Typically, NPs with zeta potentials greater than 20 mV or less than −20 mV were reported to have sufficient electrostatic repulsion to remain stable in solution^13^.

Additionally, dynamic light scattering (DLS) was used for measuring the hydrodynamic size of particles and determining agglomeration state of the nanoparticles in water and phosphate buffer (pH 7.0). DLS sizing data of the differently sized AgNPs in water and phosphate buffer (Table 1) were in close proximity indicating that there was no agglomeration of the AgNPs in Franz cell donor medium, i.e., phosphate buffer.

TEM images of the nanoparticles dispersed in phosphate buffer (pH 7.0) medium unequivocally validated the discrete existence of the AgNPs (Fig. 1D) with their dimensions almost similar to that of the synthesized ones (Fig. 1C).

### *In vitro* skin permeability study

**Figure 2:**
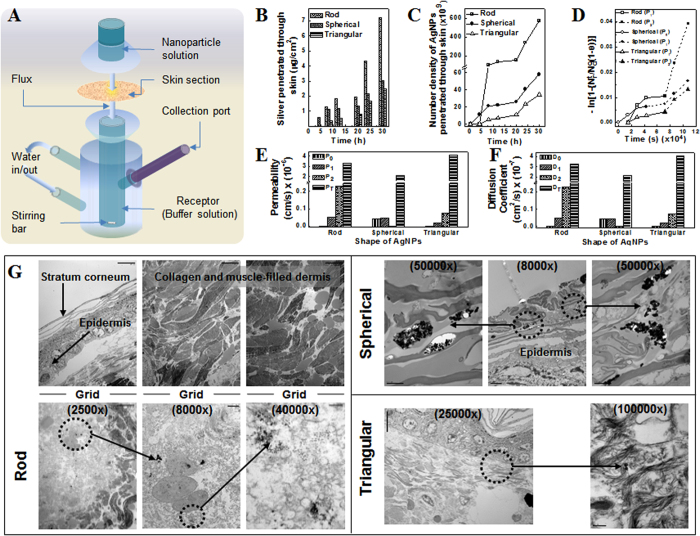
*In vitro* analysis of shape-dependent skin penetration of AgNPs. (**A**) Ultra-thin mouse skin section (surface area 1.64 cm^2^; thickness 0.7–1.0 mm) was loaded on to the Franz cell system. (**B**) Mouse skin was exposed to differently shaped AgNPs for 30 h. Amount of silver penetrated from AgNPs through mouse skin was determined and plotted as a function of time. (**C**) Number density of differently shaped AgNPs permeated through mouse skin, as a function of time in Franz diffusion cell (×10^9^). (**D**) Graph of 
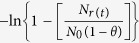
 versus *t* (s) of permeability study using mouse skin. Different phases in the permeation of differently shaped AgNPs were identified and the slope of linear fit of the different phases of permeation β_*(t)*_ (*t* = 0 for initial stage and 1, 2 for successive phases, respectively) was employed to determine the corresponding experimental permeability at initial and successive phases of permeation (*P*_*t*_) (*t* = 0 for initial stage and 1, 2 for successive phases, respectively). (**E**) Experimental and theoretical permeability coefficients of differently shaped AgNPs (×10^−6^). Experimental permeability coefficients are denoted as (*P*_*t*_) (*t* = 0 for initial stage and 1, 2 for successive phases, respectively). Theoretical permeability is represented as *P*_*T*_. (**F**) Using the permeability values, experimental diffusion coefficients of AgNPs through mouse skin (*D*_*t*_) (*t* = 0 for initial stage and 1, 2 for successive phases, respectively) associated with different phases of permeation were calculated and plotted along with theoretical diffusion coefficients. (**G**) After 30 h of exposure to differently shaped AgNPs skin tissue samples were subjected to TEM imaging to the locate of the AgNPs (dotted circles) penetrated in the skin. Tissue sections maintained good morphology throughout the experiments. This was assessed by the presence of a stratum corneum (SC) layer (approximately 10–15 μm), a compact viable epidermis (approximately 60–80 μm), and a collagen- and muscle-filled dermis. No major differences were observed in skin samples treated with differently shaped AgNPs, except that SC layer was found absent in tissue treated with SNPs. Scale bar = 10 μm (2,500×), 2 μm (8,000×), 300 nm (40,000×), 200 nm (50,000×), and 150 nm (100,000×).

*In vitro* permeability study of differently shaped AgNPs through skin was carried out using Franz diffusion cell system (Fig. 2A). The amount of silver permeated through skin to receptor cell at different time intervals was measured using inductively coupled plasma-mass spectrometry (ICP-MS) (Fig. 2B). The reported results are the average of data obtained from three to five independent sets of experiments. The standard deviations were within 5% of the average value. AgNPs showed shape-dependent permeation through skin; the permeation increased with respect to time. Interestingly, for RNPs and TNPs a lag time of 8 h was observed before silver could be detected in the receptor fluid (Fig. 2B,C). Sudden increase in the skin penetration rate of RNPs was observed after 12 h, compared to TNPs and SNPs (Fig. 2B). After 12 h the amount of silver penetrated from RNPs, SNPs and TNPs through unit area of skin was 1.82, 1.17 and 0.52 μg/cm^2^, respectively. At 30 h, TNPs showed the lowest penetration rate of 2.47 μg/cm^2^; whereas, the penetration rates for SNPs and RNPs were recorded as 3.05 μg/cm^2^ and 7.22 μg/cm^2^, respectively.

### Normalization of differently shaped AgNPs penetrated through skin

To account for the difference in silver loading or density of AgNPs due to change in atomic/lattice alignment with shape and size, the amount of differently shaped NPs penetrated through skin was normalized with respect to the number density of different AgNPs, i.e., total number of AgNPs present in the Franz cell receptor cell at a given time. Density of dried silver nanoparticles was found to be 1.32 g/cm^3^, while that of RNPs and TNPs were 1.51 and 10.49 g/cm^3^, respectively. Spherical silver nanoparticles (generally with a cubooctahedral or multiple-twinned decahedral or quasi-spherical morphology) predominantly have {100} facets along with small percentage of high-atom-density {111} facet, while in case of the rod-like silver nanoparticles (e.g., pentagonal rods), side surfaces are bound by {100} and the ends by {111} facets^5^. Therefore, silver loading in RNPs are expected to be higher than in SNPs. On the other hand the basal plane of a truncated triangular nanoplate with face-centred-cubic lattice structure (fcc) is a high atom density {111} surface. This results in comparatively high loading of silver in TNPs similar to the density of pure fcc silver. Considering the SNPs, RNPs and TNPs as sphere, cylinder and planer equilateral triangular plate, respectively, volume of one NP of each shape was calculated using their average dimensions as determined through TEM image analysis. The calculated volume of one RNP, SNP and TNP was 1.57 × 10^−17^, 6.54 × 10^−17^ and 1.13 × 10^−17^ cm^3^, respectively. Total volume of differently shaped AgNPs permeated at time *t* (*V*_*t*_) was calculated by dividing amount of silver permeated at time *t* (measured using ICP-MS) from each shape of NPs with respective density (*σ*) of the NPs. Number density of AgNPs, (defined as the total number of AgNPs present in the entire receptor cell at a given time), permeated at time *t* was calculated by dividing volume of all particles permeated at time *t* (*V*_*t*_) with volume of one particle. Number of AgNPs in unit volume of the receptor cell was not calculated as receptor volume may vary depending on the shape and size of the receptor cell of the Franz cell used; whereas the total number of nanoparticles permeated through a given area of skin of given thickness at a given time will be independent of receptor cell dimensions. The number density of differently sized AgNPs in receptor fluid at different time intervals is shown in Figure 2C. Number density of NPs in receptor fluid increased with time. Though there was a lag time of penetration for RNPs its number density in receptor fluid increased steeply after 12 h and thereafter always remained higher than the number densities of other two shapes of NPs. Number of SNPs in receptor fluid was always higher than that of TNPs. The maximum number density of RNPs was 5.68 × 10^11^ at the end of 30 h. Number density of SNPs and TNPs in receptor fluid after 30 h were 5.82 × 10^10^ and 3.43 × 10^10^, respectively. Obtained results clearly suggest shape-dependent skin penetration of AgNPs.

### AgNPs have multiple permeability phases through mouse skin

Figure 2D shows the graph of 
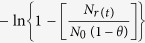
 versus *t* (in s) of *in vitro* permeability study using mouse skin, where *N*_*r*(*t*)_ denotes AgNP number density in the receptor fluid at time *t*, *N*_*o*_ is the initial number density in donor cell (at *t* = 0) and 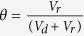 where *V*_*d*_ and *V*_*r*_ represent volume of AgNP suspension in donor and receptor buffers in the donor and receptor cells, respectively. The slope of linear fit of this plot (*β*) is related to permeability coefficient (*P*, in cm/s) and membrane (skin) surface area (*A*, in cm^2^) as 
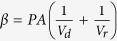
. By carefully analyzing the graph (Fig. 2D), different phases in the permeation of differently shaped AgNPs were identified and *β*_*(t)*_ (*t* = 0 for initial stage and 1, 2 for successive phases, respectively) was employed to determine the corresponding experimental permeability coefficients at initial and successive phases of permeation (*P*_*t*_) (*t* = 0 for initial stage and 1, 2 for successive phases, respectively). Using the experimental permeability values, experimental diffusion coefficients of AgNPs through mouse skin (*D*_*t*_) (*t* = 0 for initial stage and 1, 2 for successive phases, respectively) associated with different phases of permeation were calculated from the equation *D* = *Pd* where *D* is the diffusion coefficient and *d* is the thickness of the membrane.

Theoretical diffusion coefficients (*D*_*T*_) of the AgNPs were calculated using Stokes–Einstein equation considering water as medium in donor and receptor cells. Theoretical permeability coefficients (*P*_*T*_) of the AgNPs were determined using corresponding *D*_*T*_ values. For each shape of AgNPs two phases in the permeation through skin were observed. The initial and second permeability coefficients (in cm/s) of SNPs through rat skin [4.32 × 10^−8^ (*P*_0_) and 4.59 × 10^−8^ (*P*_*1*_)] were very close indicating uniform permeation of the NPs through skin. Due to initial lag time there was no initial permeability phase for RNPs and TNPs. However, unlike SNPs a third permeability coefficient (*P*_*2*_) was obtained for RNPs and TNPs. The second permeability coefficients (*P*_*1*_, in cm/s) of RNPs and TNPs were 4.75 × 10^−8^ and 1.95 × 10^−8^, respectively. The permeability coefficient of these two shapes of NPs increased in the later permeation stage. For RNPs the increase was by one order of magnitude (*P*_*2*_ = 2.29 × 10^−7^ cm/s) while the derived *P*_*2*_ value for TNPs was 7.16 × 10^−8^. It is noteworthy that the permeability coefficient of RNPs was much higher than the permeability coefficients of other two shapes of NPs. All experimentally obtained values of permeability coefficients were found to be lower than *P*_*T*_ (Fig. 2E). Diffusion coefficients (*D*) of AgNPs through mouse skin were also derived (Fig. 2F). Similar to permeability coefficient, two respective diffusion coefficients were obtained for SNPs (*D*_*0*_ and *D*_*1*_) and two each for RNPs and TNPs (*D*_*1*_ and *D*_*2*_). SNPs showed initial diffusion coefficient of (*D*_*0*_) 4.3 × 10^−9^ cm^2^/s and latter of (*D*_*1*_) 4.59 × 10^−9^ cm^2^/s. RNPs showed diffusion coefficient of (*D*_*1*_) 4.8 × 10^−9^ cm^2^/s and later of (*D*_*2*_) 2.3 × 10^−8^ cm^2^/s whereas for TNPs *D*_*1*_ was 1.9 × 10^−9^ cm^2^/s and *D*_*2*_ was 7.2 × 10^−9^ cm^2^/s. Similar to permeability coefficient, theoretical diffusion coefficient [*D*_*T*_] was derived and compared with experimental values (Fig. 2F). All experimentally obtained values of diffusion coefficient were found to be lower than *D*_*T*_as expected.

To further understand the shape-dependent skin penetration of AgNPs, the mouse skin samples from the Franz diffusion cell system (after 30 h of exposure to differently shaped AgNPs) was subjected to TEM imaging. For each shape of AgNPs ultra-thin sliced tissue sections (or grids) in the vertical direction from skin surface to inner dermal layers were observed under TEM. Thus, the main aim was to determine the penetration behavior (accumulation) of silver nanoparticles as a function of skin depth, i.e., to determine the depth of penetration of differently shaped AgNPs. Representative TEM images of grids used for RNPs were shown in Figure 2G. TEM visualization on ultra-thin sliced tissue sections showed that almost all of the skin pieces used for the penetration studies maintained good morphology throughout the experiments. It was assessed by the presence of a stratum corneum (SC) layer (approximately 10–15 μm), a compact viable epidermis (approximately 60–80 μm) and a collagen- and muscle-filled dermis (in the vertical direction from skin surface to inner dermal layer) (Fig. 2G). No major differences were observed in the skin samples treated with differently shaped AgNPs. TEM images did not show signs of NP accumulation in the follicles. TEM analysis further revealed shape-dependent skin penetration and accumulation of differently shaped AgNPs in the different layers of skin indicating their penetration through the intercellular pathway. TNPs were detected in the SC region at the depth of approximately 10 μm whereas SNPs were observed in a viable epidermal layer at a depth of 14.9–19.9 μm which indicated that both TNPs and SNPs could not penetrate through the dermal-epidermal junction into the underlying dermal layers after 30 h of treatment (Fig. 2G). On the other hand RNPs were observed in the dermal layer at a depth of 244 μm which clearly indicated their high penetration ability through the dermal-epidermal junction (Fig. 2G).

### *In vivo* skin permeability study

**Figure 3:**
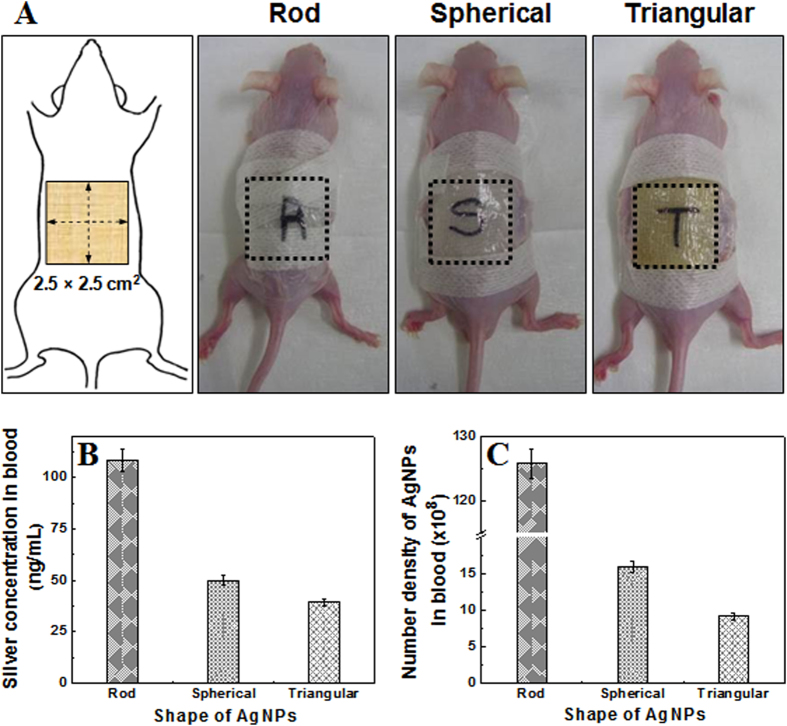
*In vivo* skin penetration analysis of three differently shaped AgNPs on hairless mice after 5 days of topical treatment. Rod-shaped, spherical and triangular AgNPs were topically applied onto the back of the mice covering an area of 2.5 × 2.5 cm^2^ (**A**). After 5 days, amount of silver in the blood samples collected from their hearts were measured using ICP-MS, and the concentration of silver (**B**) and number density of differently shaped AgNPs in blood (×10^8^) (**C**) were calculated and plotted against the different shapes of AgNPs. No significant changes in behavior, health, or treated skin areas were observed in the animals during the experiment (data not shown).

An *in vivo* study using SKH-1 hairless mice (Fig. 3A) showed varying skin penetration rates of differently shaped AgNPs in ICP-MS analysis, when systemic circulating blood samples were analyzed after five days of treatment (Fig. 3B). Corresponding to the *in vitro* data, RNPs showed the highest concentration in blood 108.57 ± 5.43 ng/mL; whereas concentration of silver permeated from SNPs and TNPs in blood were 50.00 ± 2.50 ng/mL and 39.29 ± 1.96 ng/mL, respectively (Fig. 3B). Concentration of silver (permeated from differently shaped NPs) in blood was normalized with respect to the number density of different AgNPs. Similar to *in vitro* findings, the maximum number density of NPs in blood was found for RNP-treated mice (125.79 ± 2.2 × 10^8^), followed by SNP-treated (15.84 ± 0.79 × 10^8^) and TNP-treated (9.08 ± 0.45 × 10^8^) mice (Fig. 3C).

## Discussion

The follicular penetration pathway has been reported as an “ideal” route for NPs to penetrate through skin, as follicular pores act as an excellent storage site and offer the shortest route to enter the viable skin layers^6^,^10^,^11^,^12^. These deposited NPs cannot be removed by natural desquamation, textile contact, or body wash^6^; however, some NPs can be removed by hair growth or sebum production. Earlier studies demonstrated that metal NPs^10^,^11^ (7–20 nm) and polystyrene NPs^14^ (20–200 nm) can penetrate skin through the follicular pathway. Honeywell-Nguyen *et al.* demonstrated the permeability of elastic (100–150 nm) and non-elastic particles via follicular pathway in epidermal and SC layers, respectively^12^. However, our results clearly demonstrated shape-dependent skin penetration of AgNPs through different layers of skin indicating skin penetration of AgNPs through intercellular pathway (Fig. 2G). Nevertheless, further investigations of TEM images at earlier time frames would be necessary to elucidate these facts in more detail.

Results obtained in this study demonstrate different skin penetration rates and depth-of-penetration of rod-shaped, spherical, and triangular AgNPs. In this regard, follicular penetration pathways cannot discriminate skin penetration rates of differently shaped NPs, as the follicular opening diameter is too large (26 ± 1 μm) as compared to the average sizes of AgNPs (45–50 ± 10 nm). Thus, if the follicular penetration pathway plays a central role in mammalian skin absorption, the rates of skin penetration of all the differently shaped AgNPs should have been similar, as they would all have accumulated in the follicular pores at the same time prior to their absorption through dermis.

Although mice generally have a higher density of follicles (658 ± 38 hair follicles/cm^2^) than humans or pigs, they still only cover a small area of the skin’s surface, which could limit the skin penetration rate via the follicular penetration pathway. This could be one of the reasons why we have observed the skin penetration of NPs in hairless mice through the intercellular pathway.

Thus, in a real world environment, the mode of skin penetration of AgNPs in topical applications may vary, depending on the part of the body to be treated, and the gender and ethnicity of the human population, as the density of hair follicles drastically varies in all of the above conditions. Our data suggested that the intercellular penetration pathway, and not the follicular penetration pathway, may have played a central role in the shape-dependent penetration of AgNPs through the lipid matrix between corneocytes.

*In vivo* results demonstrated penetration of AgNPs in blood capillaries; whereas *in vitro* analysis showed skin penetration of rod-shaped, spherical, and triangular AgNPs until the dermal, epidermal, and SC layer, respectively. These dissimilarities can be explained by the exposure period taken for *in vitro* (30 h) and *in vivo* (five days) analysis. In the *in vivo* analysis, a long exposure period of five days might have given sufficient time to AgNPs to slowly penetrate through the SC, epidermal, and dermal layers of skin and enter the blood circulatory system. In addition, AgNPs in the *in vivo* analysis might have received an additional advantage of actual physiological conditions, since membrane pore size may vary due to body movements, stretching, temperature fluctuations, inbuilt blood pressure, etc.

Skin penetration rates and depth-of-penetration play significant roles in determining the therapeutic potential of topical agents and their systemic toxicity^15^. Skin penetration enhancers have been routinely used in transdermal drug formulations, so that NPs could pass through SC (containing dehydrated matrix/dead keratinocytes embedded in lipid matrix), epidermal, and collagen/muscle-filled dermal layers^8^,^16^. At the same time, however, safety evaluation issues have also been considered, as NPs entering the deeper sections of skin (dermal layers) can enter the blood circulatory system^17^,^18^. In this regard, the accumulation of AgNPs in the deeper sections of skin can cause severe systemic toxic effects. Silver itself is non-toxic to humans within the reference dose [i.e., oral reference dose (*R*_*f*_*D*) = 5 × 10^−3^ mg/kg-day]^19^. Overconsumption of silver, however, may lead to argyria, which results in permanent blue-grayish pigmentation of the skin, eyes, and mucous membranes^20^,^21^. In addition, AgNPs can enhance the efficacy of transporting toxic silver ions to targeted locations in the human body.

Systemic toxicity can be caused by rapid accumulation of NPs at capillary/lymphatic junctions in the dermal layer^22^, membrane pores/legand-mediated endocytosis, and physically breached leaky endothelium^23^. Rapidly penetrating NPs could circumvent macrophage-mediated immunological responses and can enter the blood circulatory system^24^. In contrast, slow penetration offers better efficacy of NPs against the infected cells and provides adequate time for the body’s immune system to detoxify NPs through phatocytosis.

Our results demonstrated the highest penetration capabilities of RNPs and their accumulation in the dermal layer. This suggested the possibility of systemic toxicity and unwanted cosmetic side-effects, such as argyria by the accumulation of silver ions in the body^25^,^26^. The slow penetration capabilities of TNPs, compared to RNPs and SNPs, could be due to the presence of the top basal plane with predominant {111} facets in TNPs, compared to predominant {100} facets in RNPs and SNPs^5^. Earlier reports demonstrated the highest degree of bactericidal activity of silver TNPs against gram-negative bacteria *E. coli*^5^,^27^,^28^. Considering the slow penetration capability and shape-dependent bactericidal activity of AgNPs, triangular NPs could be an ideal candidate for topical applications, compared to the rod-spahed and spherical AgNPs. Silver TNPs, due to their slow penetration capabilities, can reduce systemic toxicity and increase antibacterial efficacy at lower dose levels^5^. Future investigations into the optimization of shape-dependent doses and the time frames of exposure of AgNPs in different animal models could facilitate better understanding of systemic toxicity induced by AgNPs. Although NPs are mostly preferred for their large surface area, smallness should not be a core goal, as the physicochemical properties of NPs can be efficiently utilized in topical antimicrobial formulations. We hope that our proof-of-concept study on shape-dependent skin penetration and bactericidal action could facilitate a new paradigm for understanding NPs while developing an ideal antimicrobial transdermal drug formulation. Furthermore, this proof-of-concept can be also used to characterize other metallic nanoparticles that exhibit shape-dependent therapeutic efficacy of potential interest.

## Methods

### Synthesis of AgNPs

Seed solution containing 4–5 nm silver nanoparticles was prepared by reducing 1 mL of 10 mM AgNO_3_ with 1 mL of 100 mM NaBH_4_ in the presence of 1 mL of 10 mM sodium citrate and 36 mL of fresh deionized water. The seed solution was aged for 2–4 hours before it was used to prepare bigger sized nanoparticles.

Growth solutions (A, B and C) were prepared for the seed-mediated growth step. Initially, two solutions (A and B) contained 0.25 mL of 10 mM AgNO_3_, 0.05 mL of 100 mM NaOH, 0.05 mL of 100 mM ascorbic acid and 9 mL of a 0.075 M CTAB solution. The solution C contained 2.5 mL of 10 mM AgNO_3_, 0.50 mL of 100 mM NaOH, 0.50 mL of 100 mM ascorbic acid and 9 mL of the CTAB solution.

A larger nanoparticle was prepared by adding 1 mL of seed solution to growth solution A. After 5 minutes, 1 mL of resultant solution A was then added to solution B. Again after 5 minutes, all of the resultant solution B was added to growth solution C. After addition of solution B, solution C slowly turned yellow as time progressed. The resulting solution was centrifuged at 8,000 rpm for 20 minutes to remove the excess CTAB. The obtained silver nanospheres were re-dispersed in deionized water. The silver nanospheres had an average diameter of 40–50 nm.

Truncated triangular AgNPs were synthesized by a solution-phase method for large-scale preparation of truncated triangular NPs. To the particle growth solution (5 mL of 0.01 M AgNO_3_, 10 mL of 0.1 M ascorbic acid, 146 mL of 0.1 M CTAB, and 5 mL of silver seeds), 1 mL of 1 M NaOH was added to accelerate particle growth. The color of the solution changed from light yellow to brown, red, and green within a few min. The solution was aged at 21°C for 12 h, 35 °C for 5 min, and 21 °C for 24 h. The color of the aged solution changed from green to red. The NPs were centrifuged at 2,100 × g for 10 min, and the supernatant was collected. The pellet was then dissolved in water, centrifuged again at 755 × g for 10 min, and the supernatant was collected. The collected supernatants contained the truncated triangular NPs.

Rod-shaped AgNPs were synthesized by first preparing a 4 nm seed solution (a 20 mL solution with a final concentration of 0.25 mM AgNO_3_ and 0.25 mM trisodium citrate in water) 2 h prior to synthesis. A solution of 10 mM NaBH_4_ (0.6 mL) was added all at once with vigorous stirring for 30 sec. A solution containing 0.25 mL of 10 mM AgNO_3_, 0.5 mL of 100 mM ascorbic acid, and 10 mL of 80 mM CTAB was prepared, followed by the addition of 0.06 mL of 4 nm seed solution. Finally, 0.01 mL of 1 M NaOH was added and gently shaken just enough to mix the NaOH with the rest of the solution to increase the yield of rod-shaped NPs. Within 1–10 min, the color of the solution changed from red to brown, and then to green. The solution contained a mixture of rod- and spherical-shaped NPs. The rod-shaped NPs were collected by centrifugation at 514 × g for 6 min, and the pellet was re-suspended in water.

The resulting concentrations of synthesized AgNPs were measured by inductively coupled plasma-mass spectrometry (ICP-MS) (ELAN 6100; Perkin-Elmer SCIEX). Additionally, the synthesized NPs were characterized by UV-visible spectroscopy and energy-filtering transmission electron microscope (EFTEM) (LIBRA 120, Carl Zeiss, Oberkochen, Germany). The samples for TEM imaging were prepared by placing a drop of homogeneous suspension on the copper grid with a lacey carbon film and allowed to dry in air. Mean particle size was analyzed from the digitized images with Image Tool software. UV-visible absorption spectra were recorded with an Optizen 2120 UV-visible spectrophotometer (Mecasys, Daejeon, Republic of Korea) with a 1 cm quartz cell.

### *In vitro* skin permeability study of AgNPs using a Franz cell chamber

All animal experiments were performed according to the National Institutes of Health guidelines and were approved by the Laboratory Animal Committee of the Seoul National University (Animal Ethical Approval Protocol No. SNU-121016-2). The skin samples from 7-week old SKH-1 hairless male mice weighing 30–35 g each (purchased from Orient Bio Inc., Seoul, Republic of Korea) were used in the study. A day prior to penetration study, the mouse was euthanized with CO_2_ gas, and an abdominal skin sample was excised with an electric clipper and a razor. Special care was taken during the excision procedure so that the subcutaneous fat and tissues would not be damaged. The excised skin of the euthanized mouse was mounted in the Franz cell chamber (PermeGear; Hellertown, PA, USA) with epidermis facing the donor phase and placed between the donor and receptor phases to assess the transport of NPs that had penetrated through the skin at each time point. The surface contact area of the receptor phase with the tissue sample was approximately 1.64 cm^2^. For the receptor phase, 8 mL of receptor fluid buffer (2.38 g Na_2_HPO_4_, 0.19 g KH_2_PO_4_, and 9 g NaCl in 1000 mL of distilled water at pH 7.35) was used and maintained at 37 ± 0.5 °C, while stirring with a magnetic stirrer. Approximately 500 μL of donor fluid (phosphate buffer at pH 7.0) containing the presynthesized-NPs was added into the donor chamber. Sample (300 μl) from the receptor chamber was collected at different time interval (0, 4, 8, 12, 20, 24 and 30 h). After the collection of sample, 300 μL of fresh receptor buffer was added into the receptor chamber. Each collected sample was diluted with 4 mL of 2% nitric acid solution, and the amount of silver that penetrated through the skin was determined using ICP-MS (ELAN 6100, Perkin-Elmer SCIEX).

### EFTEM imaging of skin samples

After the analysis with the Franz cell chamber, each of the skin tissue samples was sliced into 1 × 1 × 3 mM. The sliced tissues were incubated at 0–4 °C for 2 h in a fixation buffer (2% glutaraldehyde) and washed with a cacodylate buffer three times at10–20 min intervals. The tissues were fixed again with 1% osmium tetroxide (OsO_4_) at 0–4 °C for 1–2 h, washed with a cadodylate buffer three times at 10–20 min intervals, and then stained with 0.5% uranyl acetate overnight. After overnight staining, the tissues were washed twice with distilled water and dehydrated with various concentrations of ethanol (10 min incubation each in a sequential manner in the following order: 30%, 50%, 70%, 80%, and 90% and three times in 100% ethanol). All of the excess ethanol was removed by centrifugation, and fresh ethanol was added to the tissues. The dehydrated tissues underwent a transition process by treating twice with 100% propylene oxide at room temperature for 15 min. The samples were incubated in a mixed solution (Spurr’s resin and propylene oxide in 1:1 ratio) at room temperature for 2 h on an agitator, followed by removing the supernatant by centrifugation and incubation in 2 mL of 100% Spurr’s resin overnight. The supernatant was removed by centrifugation and incubated in 2 mL of Spurr’s resin for 2 h for the embedment and infiltration of tissues. The embedded tissues were added into the resin solution and incubated at 70 °C for 24 h to be polymerized into a resin block. The synthesized resin block was fine-trimmed and sliced into ultra-thin sections (60–90 nm) with a diamond knife. The sections were mounted onto a formvar-coated grid and double stained with 2% uranyl acetate (65 °C for 45 min) and lead citrate (room temperature for 30 min). The prepared tissue sections were analyzed by EFTEM with the setting at 80–120 kV.

### *In vivo* skin permeability study of AgNPs

All animal experiments were performed according to the National Institutes of Health guidelines and were approved by the Laboratory Animal Committee of the Seoul National University (Animal Ethical Approval Protocol No. SNU-121016-2). SKH-1 hairless mice (7-weeks old, male) weighing 30–35 g each were used. For each shape of nanoparticles three mice were used. Anaesthetization was done by first mixing 20 mg/mL of Rompun (Baeyer Korea Co., Seoul, Republic of Korea) and 125 mg/mL of Zoletil 50 (Virbac Lab., Carros, France) in 2:3 ratio, washing with PBS and injecting into a leg muscle of the mouse. Prior to topical treatment of AgNPs to the mouse, CTAB-coated AgNPs (1.5 mL of phosphate buffer at pH 7.0 containing 36 μg of silver) were evenly applied onto a piece of gauze with a surface area of 2.5 cm^2^. The gauze containing AgNPs was placed onto the back of an anaesthetized mouse. A waterproof bandage was applied on top of the gauze and fastened by wrapping tape around the bandage. After five days of treatment, the treated mice were sacrificed, and 5 mL of blood was taken from their hearts. The concentration of silver NPs in blood samples were analyzed using ICP-MS. Each of 700 μL of blood samples were added into 3 mL of ≥90.0% HNO_3_ (Sigma Aldrich, St. Louis, Missouri, USA) and evaporated to dryness. The mass was redissolved in 3 mL of 60% HNO_3_ and was again evaporated nearly to dryness. This step was repeated thrice and finally the residue was re-suspended in 5 mL of 2% HNO_3_ and subjected to ICP-MS analysis.

### Calculation of permeability, diffusion coefficient and number density of AgNPs

From Fick’s law of steady state diffusion the following relations can be obtained for the present study:


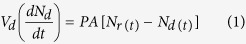


and


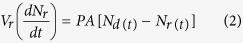


where *V*_*d*_ and *V*_*r*_ represent volume of AgNP suspension in donor and receptor buffers in the donor and receptor phases, respectively. The subscripts *d* and *r* indicate donor and receptor phase, respectively. *N*_*d*(*t*)_ and *N*_*r*(*t*)_ denote AgNP number density in the respective phases at time *t*. Permeability coefficient (expressed in cm/s) and membrane (skin) surface area (expressed in cm^2^), are denoted by *P* and *A*, respectively.

At time *t* = 0,

*N*_*r*(*t*)_ = 0 and let us assume that 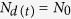


Thus at *t* = 0


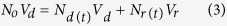


Neglecting the very low concentration of AgNPs inside the membrane the above Eq. (3) can be rearranged as:


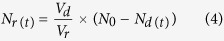


Putting Eq. (3) in Eq. (1), we get


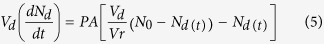



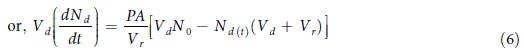



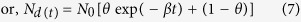


and


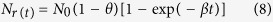


where


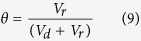



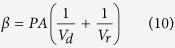


Thus, a plot of 
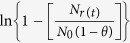
 versus *t* (expressed in s) would be a linear one with a slope of β. Experimental permeability of the AgNPs were calculated from the slope of the linear line obtained by plotting 
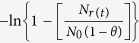
 versus *t* plot as shown in Fig. 2D.

By carefully analyzing the graph, different phases in the permeation of differently shaped AgNPs were identified and for each phase the slope (*β*) was determined. Using different *β* values corresponding permeability coefficient values of each phase was derived. The permeability coefficient of the initial permeability phase was denoted by *P*_*0*_, whereas the permeability coefficients of the successive permeability phases were represented by *P*_*1*_, *P*_*2*_ etc.

Experimental diffusion coefficients of AgNPs through mouse skin associated with different phases of permeation (*D*_*0*_, *D*_*1*_, *D*_*2*_ etc) were calculated using the corresponding permeability values in the following equation:


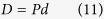


where *D* is the diffusion coefficient, *d* is the thickness of the membrane.

Theoretical diffusion coefficient (*D*_*T*_) was calculated using Stokes–Einstein equation:


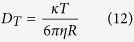


where κ is the Boltzmann’s constant (1.3807 × 10^16^ cm^2^ g s^−2^ K^−1^), *T* is absolute temperature, η is the viscosity of the medium and *R* is the radius of spherical AgNPs.

For calculating the diffusion coefficients of non-spherical NPs like rod shaped and triangular NPs, corrections were made using a radius value (*R*_*0*_) where *R*_*0*_ is defined as the radius of a sphere that has a volume equal to the volume of the non-spherical entity.

Theoretical permeability coefficient [*P*_*T*_] was calculated by putting *D*_*T*_ value in Eq. 11.

Volume of one spherical, rod-shaped and triangular NP was calculated from the following equations:


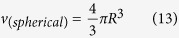



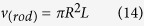



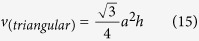


where 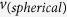, 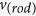, and 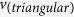 are the volume of one spherical, one rod-shaped and one triangular NP, respectively. *R* is the radius of the spherical NPs. *L* is the length and *r* is the radius of the rod-shaped NPs, while *a* is the side length of the equilateral triangular NPs and *h* is the thickness of the particle.

Total volume of differently shaped AgNPs permeated at time *t* (*V*_*t*_) was calculated by dividing amount of nanoparticles permeated at time *t* (measured using ICP-MS) with respective density (*σ*) of the nanoparticles. Further, number density of AgNPs permeated at time *t* was calculated by dividing total volume of all particles permeated (*V*_*t*_) at time *t* with volume of one particle.

## Additional Information

**How to cite this article**: Tak, Y. K. *et al.* Shape-Dependent Skin Penetration of Silver Nanoparticles: Does It Really Matter? *Sci. Rep.* **5**, 16908; doi: 10.1038/srep16908 (2015).

## Figures and Tables

**Table 1:** Zeta potential and particle size of differently shaped silver nanoparticles in water and phosphate buffer (pH 7.0) media.

Shape of AgNPs	Zeta Potential (mV)		DLS sizing data (nm)	
	Water (pH 7.0)	Phosphate buffer (pH 7.0)	Water (pH 7.0)	Phosphate buffer (pH 7.0)
Rod	31.84 ± 2.69	31.82 ± 2.67	46.0 ± 2.42	53.83 ± 3.94
Spherical	34.07 ± 8.58	36.09 ± 8.46	45.83 ± 3.33	57.83 ± 5.99
Triangle	34.59 ± 5.56	26.21 ± 5.23	63.26 ± 1.01	69.7 ± 3.06
